# Contribution of Motivational Climates and Social Competence in Physical Education on Overall Physical Activity: A Self-Determination Theory Approach with a Creative Physical Education Twist

**DOI:** 10.3390/ijerph17165885

**Published:** 2020-08-13

**Authors:** Juha Kokkonen, Arto Gråstén, John Quay, Marja Kokkonen

**Affiliations:** 1 Faculty of Education, University of Jyväskylä, 40014 Jyväskylä, Finland; juha.a.kokkonen@jyu.fi; 2 Faculty of Sports Science, University of Jyväskylä, 40014 Jyväskylä, Finland; arto.grasten@utas.edu.au (A.G.); marja.kokkonen@jyu.fi (M.K.); 3 Faculty of Education, University of Tasmania, Launceston 7248, Australia; 4 Graduate School of Education, University of Melbourne, Melbourne 3010, Australia

**Keywords:** prosocial behavior, antisocial behavior, primary school, structural equation model

## Abstract

Using a cross-sectional study design, we tested a structural equation model of hypothesized relationships among a group of variables: motivational climate in physical education (PE), students’ social competence in PE, out of-school physical activity (PA) motivation, PA intention and their moderate-to-vigorous PA (MVPA). Based on the self-reports of 363 fourth to sixth grade elementary school students (172 girls, 191 boys), the model revealed that the task-involving motivational climate in PE was linked to higher MVPA via cooperation in PE, and also via extrinsic motivation and PA intention. Ego-involving motivational climate was related to higher extrinsic motivation and amotivation, further to higher PA intention and, finally, to higher MVPA. Task-involving motivational climate was positively linked to students’ social competence markers of cooperation and empathy, and negatively to disruptiveness. Ego-involving motivational climate was positively related to disruptiveness and impulsivity, the markers of low social competence. The study showed that the motivational climate and co-operational aspect of social competence both played significant roles in students’ PA motivation, PA intention and MVPA. A pedagogical model that brings the learning of social competence relevant skills to the fore is creative physical education (CPE). Analysis of CPE is provided which highlights teaching behaviors which contribute to the students’ MVPA through motivational climates, co-operation, PA motivation and PA intention.

## 1. Introduction

Physical activity (PA) is an important factor in human health and well-being, and the physical and mental health advantages of regular PA have been extensively documented [1]. Although the benefits of being physically active are well known, a significant number of youth in many countries do not achieve the recommended amounts of PA [2] In Finland, objective measurements indicate that only 34% of elementary school students (9–15 yrs) attain the Finnish recommendations [3] of having at least 60 min of moderate-to-vigorous physical activity (MVPA) on a daily basis.

In school physical education (PE), children’s self-competence perceptions, such as perceived physical competence, have been found to affect their physical performance and behavior [4] and to be one of the most relevant contributors to PA during adolescence [5,6]. However, due to the socially constructed nature of self-competence and the PE context, which often involves interactions with peers, it is vital to understand the social aspect of perceived competence and its role in participants’ PA motivation and PA. In addition, a variety of psychosocial outcomes are related to individuals’ social competence, such as quality of life [7], psychological well-being [8,9], loneliness and social anxiety [10]. Some empirical studies have shown the positive relationship between social competence and sport/PA participation [11,12,13,14,15]. However, the role of social competence in primary school PE as part of the PA pattern warrants further exploration.

### Social Competence and Its Potential Impact on Overall Physical Activity

Social competence is a comprehensive concept including cognitive, emotional-motivational and behavioral aspects that children need for social adaption [16,17]. This study recognized the bi-dimensional nature of social competence [18] and conceptually positioned children’s social competence as consisting of two key aspects relevant to children’s social adaptation, namely prosocial behavior and lower levels of antisocial behavior [10,19,20]. Prosocial behavior refers to voluntary, socially positive behaviors benefitting others and positive peer interaction, such as comforting, helping and cooperation [21]. The other dimension of social competence is the absence of antisocial behaviors that are typically socially maladaptive and undesirable, such as impulsive and disruptive behavior [8]. In this article, we understand, like many other researchers [4] that socially competent children should exhibit cooperation and empathy and inhibit antisocial behaviors. For others, however, only prosocial behavior can be seen, indicating social competence [7] that actually falls under the umbrella concept of social functioning, together with distinct but negatively interrelated antisocial behavior [22].

In order to verify social aspects of motivation and its effects on behavior, Stump et al. [17] recommend using a bottom-up approach in terms of self-determination theory (SDT). SDT is a meta-theory involving several sub-theories which assume that human behavior consists of interaction between an individual’s environment, satisfaction of his/her needs and the level of motivational regulation [23]. It specifies three major kinds of motivational regulation on a continuum—intrinsic, extrinsic and amotivation—which vary according to the levels of self-determination, offering a continuum between more self-determined forms of motivation and more controlling forms of motivation.

The highest level of self-determination on this continuum is intrinsic motivation (enactment of an activity for its own sake, because the activity is enjoyable and interesting) [24]. At the other end of the continuum is amotivation (a lack of motivation or intention to participate) offering the lowest level of self-determination. Between these two on the continuum are four extrinsic forms of motivation, each having an instrumental effect on activity. Integrated regulation (assimilating the regulation of exercise into personal goals) and identified regulation (the outcomes of the behavior are highly valued) are both considered autonomous forms of extrinsic motivation. Introjected regulation (avoiding internal pressures or feelings of guilt) and external regulation (the activity is done because of external factors, such as rewards, constraints or fear of punishment) formulate controlling forms of extrinsic motivation. Evidence from motivation regulation studies on children and adolescents has typically revealed that autonomous motivation is positively associated with leisure-time PA [25,26], whereas controlled forms of motivation will undermine these outcomes. As an exception, Jaakkola, Washington, and Ylipiipari [27] found also a positive path from extrinsic motivation to self-reported PA of high-school students.

According to Vallerand [28], the possible positive effects of contextual factors on autonomous motivation are achieved through the satisfaction of psychological needs. The more positively contextual factors contribute to the satisfaction of the three innate needs—need for competence (need to achieve desired outcomes and to feel effective in one’s efforts), need for autonomy (need to self-organize one’s behavior and to achieve concordance between the activity and one’s integrated sense of self) and need for relatedness (need to feel connected to and accepted by significant others)—the more that autonomous types of behavior are likely to occur, which in turn, may result in positive psychosocial functioning [24].

In SDT-based studies, it is unclear which of the three psychological needs (competence, autonomy, relatedness) contributes the most to PA motivation [29]. In some SDT-based studies, social competence has been a focus, but in these studies it has been conceptualized as a social-contextual factor using only one facet (cooperation) of social competence [30,31]. Junttila et al. [27] highlighted social competence’s contributory role as it is readily identifiable and can be influenced by other social agents in social contexts, while Stump et al. [17] suggested that socially competent individuals could satisfy all three psychological needs within the context of social interaction.

In this study, the satisfaction of the innate psychological need for competence, normally conceptualized in SDT as physical competence, was approached instead using a conceptualization of social competence. This is a novel way of approaching the need for competence in PE, although PE has been seen as significant in the acquisition of values and competences relevant to students’ personal and socio-emotional development [32]. Other researchers have explored social competence in areas associated with PA: Lemonia et al. [33] found social skills (quick temperedness, disruptiveness, cooperating skills and empathy) to contribute to the prediction of perceived quality of life for recreational dancers and trainees; Gråsten et al. [12] found an individual level association between social competence and moderate to vigorous PA; Su et al. [9] indicated that the relationship between physical exercise and children’s general well-being was partially mediated by social competence; however, none employed an SDT perspective in their studies.

In addition, while the hypothesized association between innate needs, motivational regulations and PA intention have been verified theoretically via empirical studies involving secondary school PE students [24,34,35] or PA [36], far less is known about these relations in elementary school children [37,38].

Conceptualization of contextual factors in this study was realized via both task-involving and ego-involving motivational climates [39], which represent individuals’ perceptions of the stress afforded by social agents (such as teachers) on developing or demonstrating competence. Task-involving motivational climate represents hard work, co-operation, personal development and effort, whereas ego-involving motivational climate represents competition, comparisons with others, success based on ability and reward/punishment for success and failure [39,40].

Students’ perceptions of a task-involving motivational climate during PE have been found to be positively connected to psychological needs of competence, relatedness and autonomy [40], and to enhance self-determined motivation in PE [41]. The findings associated with an ego-involving motivational supportive climate have been shown to be linked with lower relatedness and self-determined motivation, and with higher amotivation [42,43]. Jaakkola et al. [27] indicated the positive path from the task-involving motivational climate via perceived competence and intrinsic motivation to high-school students’ self-reported PA but they also found that there is not always a positive link between ego-involving motivational climates and reduced self-reported PA.

The most proximal contributor of PA in this study is PA intention, which is considered a summary motivation to accomplishing a behavior [44]. In SDT-based PE studies, students’ autonomous motivation has been positively associated with intention of future PA participation [34,45] however due to mixed findings in the case of introjected regulation and amotivation, further research is needed [46]. Based on empirical findings [47,48], there is a significant positive link between intentions and future behavioral engagements, such as in PA. McEachan, Conner, Taylor and Lawton [49] approximated that intentions explain 33% of variability in PA behavior.

This study leans on the cross-contextual postulation that students’ perceptions of their psychological environment (i.e., motivational climate in PE) will contribute to their adoption of moderate to vigorous PA (MVPA) [42] via their social competence and PA motivation. As a consequence, we drew on self-determination theory [24] together with Vallerand’s [28] comprehensive model of motivation to investigate the relationship between student’s perceived motivational climates in PE, social competence in PE, out-of-school PA motivation, overall PA intention and their MVPA. Based on the aforementioned theoretical justifications, we hypothesized that social competence will explain the interconnection between contextual factors (i.e., motivational climate), PA motivation, PA intention and MVPA.

## 2. Materials and Methods

Participants were 363 (172 girls, 191 boys) elementary school children, aged between 10 and 12 years (M = 11.20 ± 0.78 years), and recruited from two public schools located in the same city in central Finland. In 2011, after receiving approval from local education authorities, all fourth- to sixth-grade children (*N* = 382) in these schools, representing a total of 5% of elementary school children in the region, were invited to participate via direct contact with the school principals. Participation was voluntary for these students, who returned signed written assents with guardian consents. No extra credit or any other benefits were rewarded for participation and withdrawal from the study was possible at any time without consequence.

Within two weeks, students in one of the schools completed electronic questionnaires via the SPSS^®^ MrInterview^TM^ software (IBM, Armonk, New York, NY, USA) in their computer class under the supervision of their PE teacher who had no access to the responses. Due to challenges accessing computer class facilities for implementing measurements across the requisite two-week period, data collection in the other school was conducted using paper-and-pencil questionnaires under the supervision of the primary investigator (PI; first author). Teachers implementing the computer-based data collection were collectively instructed by the PI so that timing and other practices related to the administration of the questionnaires were equivalent for participants. As in the other school, any misunderstandings related to completing the questionnaires were not reported by teachers. Thus, despite the slight difference in data collection protocols, in both schools, students’ information was collected through a similar process based on the same instructions and ethical principles, guaranteeing anonymity and confidentiality.

### 2.1. Measures

#### 2.1.1. Motivational Climate

Perception of motivational climate in PE was measured using the Motivational Climate in PE Scale (MCPES) [22], which consists of two subscales representing task- and ego-involving motivational climates. The individual item stem used in the measure was “In my PE class ...”. Both task-supportive (e.g., “It is important for students to try their best in PE lessons”) and ego-supportive (e.g., “It is important for students to succeed above others in PE lessons”) motivational climate dimensions consisted of four items with acceptable internal consistency, measured using Cronbach alphas (Table 1). Responses were indicated on a five-point Likert-scale ranging from strongly disagree (1) to strongly agree (5). Soini et al. [22] have demonstrated acceptable construct validity and internal consistency of the MCPES sub-scales among Finnish students.

#### 2.1.2. Social Competence

Perceptions of social competence were measured using the Multisource Assessment of Children’s Social Competence Scale (MASCS) that has been designed to fit the Finnish elementary school context, and validated [10], and consequently widely used [8,50,51] in Finland. The questionnaire administered in this study had a common stem for each item: “Using the scale below, circle the number that best describes you in PE classes.” The scale consisted of 15 items measuring four dimensions of social competence. The dimensions were cooperation (e.g., “I participate effectively in group activities”) and empathy (e.g.,”I am sensitive to the feelings of others”) for prosocial behavior, whereas impulsivity (e.g., “I have a short fuse”) and disruptiveness (e.g., “I argue and quarrel with peers”) were for antisocial behavior. Items were rated on a 4-point scale ranging from never (1) to very frequently (4). Impulsivity and disruptiveness scores were reversed. Mean scores of cooperation, empathy, impulsivity and disruptiveness were calculated and used as social competence scores for each student. Internal consistency of the social competence scale was acceptable based on Cronbach’s alphas (Table 1).

#### 2.1.3. PA Motivation

Students’ self-determined PA motivation during leisure-time was assessed with the Finnish version of the Sport Motivation Scale (SMS) [52], which was modified for the leisure PA context. The instrument has 7 subscales, comprising three types of intrinsic motivation (IM to accomplish things, IM to know and IM to experience stimulation), three forms of extrinsic motivation (identified, introjected and external regulation) and amotivation. Each dimension consists of four items. The scale had the individual item stem “I’m currently participating in leisure PA, because…?”. The students rated the reasons for their current participation in PA activities outside the school context on a 5-point Likert scale ranging from (1) describes me very poorly, to (5) describes me very well. Subscale scores were calculated for each subscale by summing a total of 12 items for intrinsic motivation and 12 for external regulation, and four for amotivation. Internal consistency of the self-determination scale was acceptable with appropriate Cronbach’s alphas (Table 1). Further, Jaakkola, Liukkonen, Ommundsen and Laakso [53] reported adequate psychometric properties for the Finnish version of the SMS.

#### 2.1.4. PA Intention

Student intention related to their future engagement in sport was quantified using the three items: “I will try to play sports during the school year”; “I plan on playing sports this school year”; and “I intend to play sports this school year” [45]. The students rated these items on a 5-point Likert scale ranging from (1) fully disagree, to (5) fully agree. The mean score of three items represented students’ PA intention and demonstrated acceptable internal consistency (Table 1).

#### 2.1.5. MVPA

The Health Behavior in School-aged Children Research Protocol was used to assess elementary school students’ overall MVPA [54]. The stem was: “In the next two questions physical activity means all activities which raise your heart rate or momentarily get you out of breath, for example, doing exercise, playing with your friends, going to school, or in school PE. Sport also includes, for example, jogging, intensive walking, roller skating, cycling, dancing, skating, skiing, soccer, basketball, and Finnish baseball.” The scale consisted of two items: “Think about a typical week for you. On how many days did you exercise for at least 60 min during which you got out of breath?” and “Think about the last 7 days. On how many days did you exercise for at least 60 min during which you got out of breath?”, that students rated using an 8-point response scale (0–7 days of the week). The mean score of the two items represented children’s total MVPA scores indicating acceptable internal consistency (Table 1).

### 2.2. Statistical Analyses

First to be examined were normal distribution, outliers and missing values of the data. Modifications due to normality or outliers were not required [55]. In total, 3.3% of values for individual items were found missing. A few missing values occurred because some children did not provide fully completed questionnaires. Little’s MCAR test (χ^2^ = 4100.326, df = 4203, *p* = 0.869) indicated that the missing values were completely random (MCAR) [33].

Next to be determined were correlation coefficients, Cronbach alphas, means and standard deviations for each variable. In order to test the factor structures of the scales, confirmatory factor analyses were conducted. A path model was implemented to test the associations between task- and ego-involving motivational climates, cooperation, empathy, disruptiveness, impulsivity, intrinsic motivation, extrinsic motivation, amotivation, PA intention and MVPA. The covariance effects of gender, school, grade, class and teacher were also analyzed.

The chi-square test (χ^2^) was applied as a test of the model’s overall goodness-of-fit. A non-significant difference between observed frequency distribution and theoretical distribution demonstrates an acceptable fit to the data. To determine the suitability of the model, the standardized root mean square residual (SRMR), the root mean square error of approximation (RMSEA), the comparative fit index (CFI) and the Tucker–Lewis index (TLI) were also examined [56]. A value less than 0.06 for SRMR is generally considered as a good model fit and a value of 0.08 or less for the RMSEA indicates a reasonable error of approximate fit [57]. For the CFI and TLI indices, values greater than 0.95 are indicative of an excellent model fit [57]. The proportions of variance were examined using squared multiple correlations (R^2^). The missing value analysis was analyzed using SPSS Version 22.0 (IBM, Armonk, NY, USA) and the path model was conducted using Mplus Version 7.11 (Statmodel, Los Angeles, CA, USA) [58].

## 3. Results

As shown in Table 1, the mean scores indicated that children’s perceptions of the task-involving motivational climate, PA intention and MVPA scored relatively highly. In turn, the perceptions of the ego-involving motivational climate, cooperation, empathy, intrinsic motivation and extrinsic motivation were moderate. In addition, the perceptions of disruptiveness, impulsivity and amotivation could be considered as low. The correlations showed that the associations between variables ranged from low negative to strong positive (−32 to 0.77). The strongest positive correlations were found between intrinsic motivation/extrinsic motivation and prosocial behavior variables. The strongest negative association was detected between empathy and disruptiveness.

Confirmatory factors analyses were implemented to test factor structures of the scales. All scales were found to have satisfactory fit indices: Motivational Climate in PE Scale (MCPES) (χ^2^ (26) = 51.53, *p* = 0.002, CFI = 0.96, TLI = 0.95, RMSEA = 0.051, CI 90% (0.03, 0.07), SRMR = 0.042), Social Competence Scale (MASCS) (χ^2^ (84) = 168.78, *p* < 0.001, CFI = 0.94, TLI = 0.92, RMSEA = 0.052, CI 90% (0.04, 0.06), SRMR = 0.053), Sport Motivation Scale (SMS) (χ^2^ (203) = 413.80, *p* < 0.001, CFI = 0.91, TLI = 0.90, RMSEA = 0.057, CI 90% (0.5, 0.07), SRMR = 0.067), and PA intention and MVPA scale (χ^2^ (4) = 4.30, *p* = 0.367, CFI = 1.00, TLI = 0.99, RMSEA = 0.014, CI 90% (0.00, 0.08), SRMR = 0.025). Based on this, the subscales provided reliable results for the path model development.

In order to test the associations between task- and ego-involving motivational climates, cooperation, empathy, disruptiveness, impulsivity, intrinsic motivation, extrinsic motivation, amotivation, PA intention and MVPA, a path model was constructed. This theorized model had all possible paths specified. The first step was to run the theorized model for the purpose of seeing which paths were not significant. Second, this model was configured by removing the non-significant paths. The model revealed an excellent model fit for the data (χ^2^ (1) = 1.171, *p* = 0.279, CFI = 1.00, TLI = 0.98, RMSEA = 0.023, CI 90% (0.00, 0.15), SRMR = 0.006).

**Figure 1:**
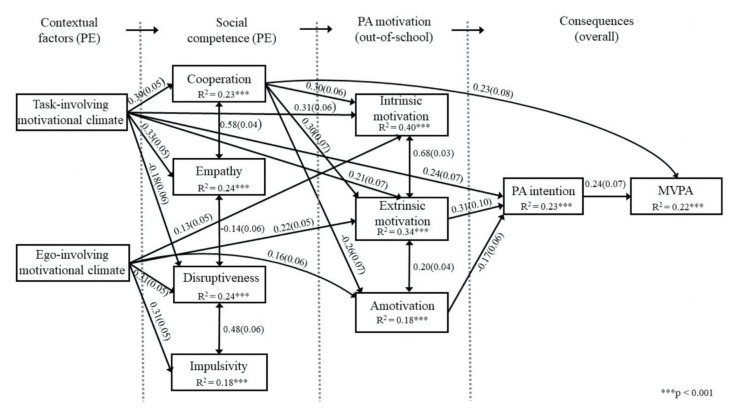
Standardized parameter estimates for hypothesized model. All paths are significant at *p* < 0.05 level.

The final model showed positive direct paths from the task-involving motivational climate to cooperation, empathy, intrinsic motivation, extrinsic motivation and PA intention, in addition to a negative direct path to disruptiveness (Figure 1). Ego-involving motivational climate was in turn positively linked with disruptiveness, impulsivity, intrinsic motivation, extrinsic motivation and amotivation. In the next column, cooperation was positively related to intrinsic motivation, extrinsic motivation and MVPA, whereas the relationship with amotivation was negative. Additionally, positive associations between the prosocial behaviors of cooperation and empathy and the antisocial behaviors of disruptiveness and impulsivity, as well as a negative relation between empathy and disruptiveness, were detected. Next, intrinsic motivation had a significant link with extrinsic motivation, and extrinsic motivation with amotivation. Amotivation was negatively related to PA intention that was further positively linked with MVPA. The model explained 18 to 41% of variance of the study variables, with the highest squared multiple correlation (R^2^) found in the intrinsic motivation variable and the lowest in impulsivity.

Six indirect paths were found. These particular paths were from the task-involving motivational climate through cooperation, extrinsic motivation and PA intention to MVPA (standardized estimate = 0.01, *p* < 0.05); task-involving motivational climate through cooperation and extrinsic motivation to PA intention (standardized estimate = 0.04, *p* < 0.01); task-involving motivational climate via cooperation and amotivation to PA intention (standardized estimate = 0.02, *p* < 0.05); task-involving motivational climate to PA intention via cooperation (standardized estimate = 0.12, *p* < 0.001); task-involving motivational climate to extrinsic motivation via cooperation (standardized estimate = 0.12, *p* < 0.001); and task-involving motivational climate to amotivation via cooperation (standardized estimate = −0.10, *p* < 0.001).

The covariance effects of gender, school, grade, class and teacher were also analyzed. A significant covariance effect of gender on MVPA (standardized estimate = 0.16, *p* < 0.01), amotivation (standardized estimate = −0.14, *p* < 0.05) and empathy (standardized estimate = −0.22, *p* < 0.001) was noticed. In other words, boys were more physically active than girls, but had lower scores in empathy and amotivation than girls. Additionally, school had an effect on amotivation (standardized estimate = −0.71, *p* < 0.01), disruptiveness (standardized estimate = −0.89, *p* < 0.01) and impulsivity (standardized estimate = −0.59, *p* < 0.01), indicating differences between schools. Grade was related with amotivation (standardized estimate = −0.37, *p* < 0.05) and cooperation (standardized estimate = −0.37, *p* < 0.05), i.e., the higher grade, the lower amotivation and cooperation scores. The covariance effect of class was significant for intrinsic motivation (standardized estimate = 0.84, *p* < 0.01), extrinsic motivation (standardized estimate = 0.88, *p* < 0.05), amotivation (standardized estimate = 0.69, *p* < 0.05) and disruptiveness (standardized estimate = 0.90, *p* < 0.05), showing significant differences between classes. The covariance of teacher had a significant effect on intrinsic motivation (standardized estimate = −0.43, *p* < 0.01), extrinsic motivation (standardized estimate = −0.58, *p* < 0.001) and empathy (standardized estimate = −0.36, *p* < 0.05), showing that the teacher, along with their personality and teaching strategies, is connected to children’s PA motivation and empathy levels.

## 4. Discussion

The relationship among perceptions of motivational climates, prosocial and antisocial aspects of social competence, PA motivation, PA intention and MVPA were explored in elementary school children aged between 10 and 12 years. This study extends previous research by using an SDT approach [59] directed by social competence in PE, which may promote transference of PA behavior to contexts outside of school. Primarily, our results demonstrate that, in addition to the motivational climates in school PE, the perceived social competence in school PE, particularly the aspect of cooperation, might be a significant source of children’s self-determined PA motivation, PA intention and MVPA.

The hypothesized sequence of relationships [24,28] were generally supported, and as contextual factors, psychological need (social competence), PA motivation and PA intention positively contributed to students’ MVPA, explaining 22% of its variance. Partly supporting previous positive findings of association between autonomous motivation and PA [25,26], we found that extrinsic motivation contributed to associations between social competence, PA intention and MVPA. The explanation for this finding may lie in the fact that our extrinsic motivation subscale included both autonomous (identified regulation) and controlling forms of motivation (introjected and external regulation), supporting the findings of Jaakkola et al. [27]. Surprisingly, there was no direct link between intrinsic motivation and PA outcomes.

There are several possible explanations for these findings. Despite the focal role of intrinsic motivation on MVPA in sport, not all the exercise activities are always intrinsically interesting. Instead of intrinsic motivation, identified regulation is identified as the main positive predictor of PA intentions in exercise activities, which may be linked to the nature of the activity [31]. Owen, Astell-Burt and Lonsdale [60] found a positive relationship between identified regulation and objectively measured PA during PE. As in our study, MVPA and out-of-school PA motivation measurements covered various PA contexts such as competitive sport, hobbies and/or free play. Hence, it is possible that identified regulation motivation overrode intrinsic motivation.

It has also been reasoned that intrinsic and extrinsic motivation might represent more or less independent, orthogonal constructs [24,61], meaning that, in our study, only extrinsic motivation was related to MVPA via PA intention. On the other hand, despite the adequate psychometric properties of the Finnish version of the SMS in high-school students [53], this scale may not clearly differentiate between intrinsic and extrinsic motivation in primary school students. In addition, the substantial correlation between intrinsic motivation and extrinsic motivation (r = 0.77) complicates the indication of the unique effects of these two variables on outcomes, which may partly explain the absence of any direct link between intrinsic motivation and PA outcomes.

One central aim of this study was to explore the role of social competence within an SDT framework. In line with the SDT assumption of the need for competence [24], cooperation as an aspect of social competence in PE positively contributed to autonomous forms of PA motivation and negatively to amotivation, explaining (together with motivational climate) high proportions of variance in intrinsic motivation (40%) and extrinsic motivation (34%). In addition, and contradicting earlier findings [29,30], a direct link occurred between cooperation in PE and MVPA, implying that, by emphasizing student practice of co-operational skills and their perceptions of their own cooperation in school PE, PA motivation in out-of-school contexts might be promoted, and further, their overall MVPA. In future research, the role of cooperation should be confirmed with detailed longitudinal research designs.

One explanation for why only cooperation (and not the other aspects of social competence) predicted overall MVPA could be the nature of single items in the social competence sub-dimensions. Cooperation included items such as “I participate effectively in group activities,” which might be more prone to influence from contextual factors and inclusion of voluntary, out-of-school learning contexts, especially when compared with the items for impulsivity (e.g., “I have a short fuse”) and disruptiveness (e.g., “I argue and quarrel with peers”). Secondly, the link between cooperation and MVPA might also have to do with the content of MVPA that the participating students reflected upon. Although we did not collect data on the sport hobbies of the participating students, there is an increasing trend in Finnish 9- to 15-year-olds to do organized sports in sport clubs where team sports, such as football, are the most popular types of sports [3]. A plausible explanation for the cooperation–MVPA link is that our participants practiced team sports where cooperation between teammates is highly adaptive and necessary. On the other hand, behaviors such as interrupting, arguing and quarreling, and making fun of other students, are typically seen as undesirable negative student behaviors by PE teachers [62]. Therefore, it seems understandable that impulsivity and disruptiveness, which PE teachers typically try to constrain through disciplinary actions in PE classes, did not predict students’ out-of-school PA motivation, PA intention or their overall MVPA.

Cooperation has been shown to have positive effects on the different motivational types [30,39,63], although cooperation has been positioned as one of the social factors affecting psychological needs, not as a psychological need in itself, which complicates the comparison of the results. However, clarification is still required to distinguish the three need satisfactions [37] and the independent contributions of each individual need to motivation [64].

Results concerning the role of a task-involving motivational climate supported previous findings in which the task-involving motivational climate has been found to be positively connected to psychological needs [30], and enhance self-determined motivation in PE [41], and to be positively correlated with intrinsic motivation and increased MVPA engagement [27], whereas results concerned with an ego-involving motivational climate have been less clear. Opposite to typical findings [42,43], there was no relationship between the ego-involving motivational climate and psychological needs in terms of co-operation or empathy, and a positive relationship was found between ego-involving motivational climate and intrinsic motivation, and ego-involving motivational climate and MVPA via extrinsic motivation.

Our findings are in line with achievement goal theory, suggesting that task- and ego-involving climates have an orthogonal relationship [65], meaning that a task-involving motivational climate is independent from an ego-involving motivational climate, which might explain why only a task-involving motivational climate was associated with cooperation and empathy. This makes sense especially in this study, as a task-involving motivational climate itself, among other things, represents cooperation and personal development.

The possible psychometric challenges related to the Finnish version of the SMS may explain why an ego-involving motivational climate was positively linked to intrinsic motivation and further MVPA through extrinsic motivation. Jaakkola et al. [27] also found that there is not necessarily always a positive link between ego-involving motivational climates and reduced self-reported PA. According to Roberts, Treasure and Kavussanu [66], task-involvement in PE might prevent students’ motivation towards PA participation being negatively contributed towards by ego-involvement. Furthermore, the trans-contextual perspective flavored with Finnish youth sport culture needs to be born in mind, as this may influence the relationship between students’ perceptions of their ego-involving motivational climate in PE and their intrinsic PA motivation in out-of-school contexts. According to Blomqvist, Mononen, Konttinen, Koski and Kokko [67] altogether 62% of 9- to 15-year-olds participated in sport club activities, this being the highest among 11-year-olds (71%), and the bulk of these youth (76%) participated in competitive sports. Due to participants’ possible competitive backgrounds, the positive link between ego-involving motivational climate in PE and out-of-school intrinsic motivation is understandable, as for these participants, social comparison and continuing rivalry might be sources of pleasure and fun in various PA contexts.

From a pedagogical perspective, our findings add to the discussion of which independent psychological need contributes the most to self-determined motivation [9,29], and how to design teaching strategies and practices that enhance social competence and increase self-determined motivation and MVPA. Despite different autonomy-supportive motivational environment designs stressing self-improvement or competence enhancing strategies implemented through well-structured environments [30] the primary source of self-determined motivation could not be represented by competence, autonomy or relatedness alone [29,30]. As a consequence, following the suggestion expressed by Sun and Chen [9], it might be fruitful to develop a more comprehensive pedagogical framework in PE, supportive of SDT, which addresses both the learning context and curriculum. This could help identify more factors and expand mechanisms by which individuals’ need satisfaction and self-determined motivation, and overall MVPA could be enhanced.

### 4.1. Pedagogical Links between Learning Context, Social Competence, PA Motivation and the Consequences for MVPA

A number of pedagogical models in PE have sought to improve teaching and learning in ways supportive of SDT. Vasconcellos et al. [68] mentioned Teaching Games for Understanding [69,70], and Sport Education [71] as exemplars of pedagogical models “that have been shown to have positive impact on students’ motivation” (p. 14). Other comprehensive pedagogical models exist which emphasize students’ motivation by also drawing on taking personal and social responsibility in PE [29]. Amongst these is creative physical education (CPE) [12,72,73,74,75], so called because it involves students in teams creating a game that can be played in a sport season where team improvement is the focus. 

To engender a task-involving motivational climate, CPE can be expressed through the TARGET framework [76], wherein teachers design the pedagogical context in terms of meaningful tasks (T), shared authority (A), recognition (R), meaningful grouping (G), individual evaluation (E) and a sufficient amount of time (T) for learning. 

Here, CPE begins with meaningful grouping (G). The key here is being a team member: this is the most meaningful aspect of PE for students, and yet it is often downplayed by teachers. CPE is built around a team-based pedagogy, where the teams are carefully selected by the teacher and stay together for the duration of the unit. Team-based pedagogy provides a central explanatory rationale for CPE, “to explain why the behavior is truly worth the students’ effort” [77]. Being a team member is scaffolded by a series of team member levels adapted from the work of Hellison [78], through 1 (disruptive), 2 (generally cooperative), 3 (contributes when asked by name), 4 (contributes without being asked) to 5 (leads others). These levels articulate behavioral expectations, but they are not simply controlling features policed by the teacher; instead, they communicate how to be a team member, offering recognition (R) for team-supportive behavior. The accompanying rationale is that the more team members in a team operate at the higher levels, the better the teamwork, and the better the performance of that team.

Crucially, these team member levels are not introduced and managed by way of teacher control, “through messages that are rigid, evaluative and pressure inducing” [77]. They are introduced, commonly, using role play, and then team members are asked to self-assess or individually evaluate (E) their performance in a lesson against the levels, giving examples as evidence and with the emphasis on improvement in the next lesson—for the sake of the team. They self-monitor this improvement through record keeping. In this sense, these behavioral expectations are communicated via informational, noncontrolling language, and managed by each student in a way which emphasizes autonomous self-regulation.

Individuals grow together as team members through the meaningful tasks (T) they are charged with performing. The first is for each team to create a game. A feature of team-based pedagogy applied in the development of games is the use of criteria, for example, the game must be: (a) fun to play with another team, include skills A and B (such as movement skills); (b) able to be played without a designated referee or scorer (so that students are all players, the teacher is not positioned as the sole adjudicator and more than one game can occur at once); (c) inclusive of all team members playing all the time (no one is excluded); (d) inventive in the use of equipment provided (each team has the same equipment which can be shared); (e) contained within the space provided (often half a basketball court); and (f) able to be completed within ten or so minutes (this is so that during the season there is ample time in lessons for analysis and practice). These criteria are designed to enable shared authority (A): the game is not the teacher’s game but is very much the creation of the students.

Game creation occurs via a round robin process between teams, which may take three or more lessons. Teams initially draft their first imperfect attempt using the criteria. Teams are then paired, and each team has the opportunity to teach their draft game to the other team (a process which is scaffolded) and play it until the other teams understand it. Immediately following this, teams meet to discuss the other team’s draft game and to determine feedback against the criteria. This team feedback, which is consensus-driven individual evaluation (E), is recorded in a written form and passed to the other team. In this way, recognition (R) for achievement and provision of feedback is enabled via shared authority (A). At the beginning of the next lesson, teams revisit this feedback and use it to further develop their game, this time being paired with a different team to teach and play their improved game, and repeat the feedback cycle. At the conclusion of the round robin, the design of each team game contributes to the development of a class game, a process managed by the teacher. The teacher’s draft of a class game is then the focus of further cycles of review and improvement until it is ready for inclusion as the focus of a season of games. Through the season, the emphasis is on evaluation (E) of team performance and the meaningful task (T) of designing practice activities which support team improvement.

As a unit of work in PE, CPE may extend for many weeks, a sufficient amount of time (T) is required as teams grow while creating games, participating in a season and striving for improved performance: performance which is both individual (team member) and collective (team). The game creation process, and the season which follows, is designed to allow time for self-paced learning, supported socially within teams.

The task-involving motivational climate that is engendered through this pedagogical model creates a learning context that emphasizes the psychological need for not only physical competence but social competence. Being a team member combined with a team-based pedagogy creates a mastery orientation, fostering “feelings of belongingness” and cooperation [39]. The importance of cooperation is highlighted in such a learning context, which contributes to engagement in the activity for its own sake (intrinsic motivation), because it is enjoyable and interesting. Further, autonomous forms of extrinsic motivation are visible in that the outcomes of the behaviors are highly valued (identified regulation) and assimilated into personal goals (integrated regulation). Elements of introjected regulation and external regulation may also be present, especially where the team is concerned, but the shared authority enabled by the team focus is very different to that experienced when the teacher adopts a controlling motivating style [77].

The cooperative effort required in such autonomy-supportive learning contexts, concomitant with encouragement of autonomous forms of motivation, suggests the possibility of increasing MVPA in PE as well as in contexts beyond PE. While these broader contexts may be very varied—some competitive, others free play, and also including hobbies—they share a need for social competence expressed through cooperation in situations characterized by autonomous motivation.

### 4.2. Limitations and Strengths

The present study has some methodological limitations. First, we relied on cross-sectional data, and therefore no causal conclusions can be drawn from the identified associations between variables. In the future, longitudinal or experimental data are needed to demonstrate causality. Secondly, all measures were self-reports. Our study would have benefitted from the combination of both self-reports and the reports given by other informants, such as PE teachers or elementary school students’ parents. As for MVPA, measures that are more objective would have been valuable, too. Thirdly, there is always a possibility that variance associated with the survey format affected students’ responses due to slightly different data collection protocols (online questionnaires vs. paper-and-pencil questionnaires) between schools. Finally, a covariance effect did occur, even if the data were collected from two schools located in the same, typical middle-size city having equal access to local community PA facilities. Thus, our results cannot be generalized to other fourth–sixth-graders in Finland.

This present study, incorporating a relatively high number of participants, extends previous investigations taking an SDT approach by introducing social competence as an addition to physical competence, in consideration of the psychological need for competence. In this study, the introduction of social competence illuminated the circumstance where those pedagogical practices which are positively related to social competence, organized (mainly) by the PE teacher, may influence a student’s out-of-school PA motivation and MVPA. With this insight comes an awareness of the importance of pedagogical innovations which support learning in the area of social competence, particularly in cooperation.

### 4.3. Future Directions

The connections made through this study highlight the need for more research investigating social competence as a contributor to the psychological need for competence, and its impact on motivation. This research should impact the instructional behaviors of teachers and the pedagogical practices they design. Broader implementation of pedagogical models like CPE can support this work, as they take strides in responding to the need for pedagogical change identified through research driven by theories of motivation such as SDT. In this way, this present study agrees with the findings and suggestions made via the recent literature review conducted by Vasconcellos et al. [68], which pointed to “a relative lack of objective measures of social support in the literature”, necessitating “more research to understand how observable teacher and peer behaviors influence motivational processes and outcomes” (p. 14). The emphasis on peer support is central here, as promoted by CPE, supporting another finding of Vasconcellos et al. [68] “that peer support has been rarely studied in physical education”, representative of “another promising area for future interventions” (p. 14).

## 5. Conclusions

Student motivation is an important issue in the teaching of physical education. Self-determination theory is a theoretical model which supports understanding motivation, based on the idea that three basic psychological needs impact on this motivation: autonomy needs, competence needs and relatedness needs. The need for competence is commonly interpreted in physical education literature as physical competence. This article reported a study that investigated social competence as a factor in motivation. This is a new development in the application of self-determination theory in physical education. The results of this study highlight the importance of PE teachers’ use of innovative pedagogies and teaching practices, such as CPE, which involve the creation of a task-involving motivational climate emphasizing co-operation in PE lessons and units of work. This, in turn, may influence student’s MVPA via out of-school intrinsic and extrinsic motivations.

## Figures and Tables

**Table 1:** Correlations, Cronbach alphas (α), means (M) and standard deviations (SD) of the study variables.

	1	2	3	4	5	6	7	8	9	10	α	M	SD
1 Task-involving motivational climate	−										0.77	4.29	0.64
2 Ego-involving motivational climate	−0.02	−									0.82	2.76	0.98
3 Cooperation	0.40 *	−0.10	−								0.74	3.18	0.45
4 Empathy	0.34	−0.14	0.66 ***	−							0.65	3.32	0.48
5 Disruptiveness	−0.19 *	0.33 ***	−0.19	−0.32 ***	−						0.79	1.44	0.50
6 Impulsivity	−0.10	0.29 **	−0.21	−0.25	0.54 ***	−					0.74	1.78	0.55
7 Intrinsic motivation	0.50 ***	0.07	0.52 *	0.42 ***	−0.12	−0.12	−				0.92	3.80	0.74
8 Extrinsic motivation	0.36	0.19 **	0.44 *	0.32	−0.01	−0.05	0.77 ***	−			0.89	3.55	0.72
9 Amotivation	−0.08 *	0.24 *	−0.21 ***	−0.13	0.23 *	−19 *	−0.14 ***	0.07 ***	−		0.69	2.29	0.90
10 PA intention	0.36 ***	0.01	0.27	0.15	−0.05	−0.07	0.36	0.35 ***	−0.20 **	−	0.77	4.74	0.59
11 MVPA	0.21	0.03	0.29 **	0.14	−0.07	−0.13	0.30 *	0.26 ***	−0.17 *	0.30 ***	0.83	5.53	1.50

Note *** *p* < 0.001, ** *p* < 0.01, * *p* < 0.05.
